# Cardiovascular disease and amyloidosis in phase III multiple myeloma trials: a systematic review of eligibility, screening and event reporting

**DOI:** 10.1136/openhrt-2026-004240

**Published:** 2026-09-01

**Authors:** Oliver Henriques Peck, Aimee McCoubrey, John A Snowden, Ashutosh Wechalekar, Jennifer Travers, Jennifer S Lees, Mark C Petrie, Ninian N Lang

**Affiliations:** 1 School of Cardiovascular and Metabolic Health, College of Medical and Veterinary Life Sciences, University of Glasgow, Glasgow, UK; 2 NHS Greater Glasgow and Clyde, Glasgow, UK; 3 NHS Golden Jubilee University Hospital, Glasgow, UK; 4 Department of Haematology, Sheffield Teaching Hospitals NHS Foundation Trust, Sheffield, UK; 5 Division of Clinical Medicine. School of Medicine and Population Health, The University of Sheffield, Sheffield, UK; 6 University College London (Royal Free Campus), National Amyloidosis Centre, London, UK

**Keywords:** Systematic Reviews as Topic, Amyloidosis, Quality of Health Care

## Abstract

**Background:**

Cardiovascular disease (CVD) and light chain (AL) amyloidosis are important causes of morbidity and mortality in patients with multiple myeloma (MM). Additionally, MM therapies can have cardiovascular effects. However, the extent to which phase III MM trials account for underlying CVD and amyloidosis through eligibility criteria, screening and reporting remains uncertain. These elements of trial design and reporting are essential for accurate interpretation of cardiovascular safety signals.

**Objectives:**

To examine practice-changing MM trials with respect to amyloidosis and CVD eligibility criteria, assessment for cardiac amyloidosis, reporting of baseline cardiovascular characteristics and cardiovascular adverse events (CVAE).

**Methods:**

Systematic review of phase III randomised controlled trials of MM drug therapies published in English between 2015 and 2025, identified by searching MEDLINE, Embase and Cochrane Library. Eligible trials enrolled adults aged ≥18 years with MM and included ≥100 patients per treatment group; non-randomised, subgroup and abstract-only reports were excluded.

**Results:**

41 trials enrolling 20 144 participants were included. Amyloidosis was an exclusion criterion in 30 trials typically using non-specific definitions. No trial reported active screening for amyloidosis or assessment of cardiac involvement. Baseline cardiovascular investigations were common but were not used to identify cardiac amyloidosis. At least one cardiovascular exclusion criterion was present in 98% of trials, most commonly heart failure and recent myocardial infarction. Baseline cardiovascular characteristics were reported in only one trial. CVAEs were investigator-reported, inconsistently defined and not centrally adjudicated.

**Conclusions:**

Phase III MM trials frequently incorporate amyloidosis and CVD exclusion criteria. However, the prevalence of cardiac amyloidosis and other CVD within these practice-changing MM trials remains poorly defined and CVAE reporting remains suboptimal. These limitations may bias safety and efficacy outcomes and reduce trial generalisability.

**PROSPERO registration number:**

CRD420251125097.

**WHAT IS ALREADY KNOWN ON THIS TOPIC**

- Cardiovascular disease and amyloid light chain amyloidosis are common in patients with multiple myeloma, and contemporary myeloma therapies may contribute to cardiovascular toxicity. However, the extent to which phase III multiple myeloma trials account for these conditions in eligibility criteria, screening and adverse event reporting remains unclear.

**WHAT THIS STUDY ADDS**

- This systematic review highlights substantial heterogeneity in the assessment, exclusion and reporting of amyloidosis and cardiovascular disease in contemporary phase III multiple myeloma trials.

**HOW THIS STUDY MIGHT AFFECT RESEARCH, PRACTICE OR POLICY**

- Standardised cardiovascular and amyloidosis assessment and reporting may improve interpretation of cardiovascular safety and the generalisability of future myeloma trials.

## Introduction

 Multiple myeloma (MM) is a haematologic malignancy characterised by clonal proliferation of plasma cells. Cardiovascular disease (CVD) is highly prevalent in patients with MM, reflecting shared risk factors for malignancy and older age at diagnosis.^1^ Nearly 70% of patients with MM have pre-existing CVD, including heart failure (HF), atrial fibrillation, hypertension or coronary artery disease.^2^

Light chain (AL) amyloidosis is estimated to affect approximately 10%–15% of patients with MM and frequently involves the heart, which is the dominant determinant of prognosis.^3–5^ Cardiac AL amyloidosis may be clinically silent in early stages but often progresses to HF, arrhythmias, conduction disease and sudden cardiac death.^6^ Early cardiac involvement may be under-recognised without systematic evaluation, particularly in patients with overlapping cardiovascular (CV) comorbidity or treatment-related symptoms.

Clinical trials of MM treatments frequently aim to exclude patients with systemic or cardiac AL amyloidosis. However, the extent to which active protocol-mandated assessment for amyloidosis is performed during the trial screening phase may be suboptimal. Consequently, patients with unrecognised cardiac amyloidosis may remain enrolled while those with overt or higher-risk phenotypes may be selectively excluded, obscuring amyloid-specific CV safety and treatment efficacy signals and limiting trial generalisability.

Many contemporary MM therapies are associated with CV adverse effects^7–9^ including thrombosis, hypertension, HF and arrhythmia.^10–12^ We previously showed that, in solid cancer drug trials, heterogeneity in CV eligibility criteria and reporting of baseline CV characteristics and CV adverse events (CVAEs) limit the interpretation of findings in relation to CV risk and outcomes.^13^ Whether similar patterns exist in MM trials remains unclear, but this is particularly relevant given the high prevalence of CVD and the additional complexity introduced by occult AL amyloidosis.

We conducted a systematic review of the design and reporting of phase III randomised controlled trials of MM drug therapies. Our objectives were to examine trial eligibility criteria relating to amyloidosis and CVD, the use of protocol-mandated CV investigations and screening for cardiac amyloidosis, the reporting of baseline CV characteristics and CVAE reporting.

## Methods

This systematic review of trial design and reporting was registered with the International Prospective Register of Systematic Reviews (PROSPERO; CRD420251125097)^14^ and used the Preferred Reporting Items for Systematic Reviews and Meta-Analyses (PRISMA) guidance. We used Population, Intervention, Comparison, Outcome (PICO) criteria for inclusion (online supplemental table 1). The objective was to characterise trial design and reporting practices rather than evaluate treatment efficacy. Therefore, formal risk-of-bias assessment tools were not applied. Patients and the public were not involved in design, conduct or reporting of this systematic review.

### Search strategy

The search was conducted in MEDLINE, Embase and Cochrane on 14 May 2025. All publicly available trials to the date of extraction were eligible. Search terms are included in the online supplemental table 2. Duplicates were removed. Articles were identified by two independent reviewers (OHP and AMc), with disagreements resolved by consensus with a third reviewer (JSL).

### Study eligibility criteria

A systematic search identified MM drug trials in adults (≥18 years old) and published from 1 January 2015 to the date of extraction. We included phase III randomised controlled trials enrolling ≥100 participants with results published at the time of extraction. Eligible trials compared the intervention of interest against placebo or an active control. Both open-label and double-blind randomised trials were eligible. We excluded stem cell transplantation and chimeric antigen receptor therapy, as well as non-randomised controlled trials, meta-analyses, review articles, commentaries, cost-effectiveness analyses, published abstracts and patient-reported outcome analyses. Where multiple publications reported the same cohort, the original publication was used.

### Outcomes

Key trial characteristics, eligibility and exclusion criteria relating to CVD and/or amyloidosis were extracted. Screening and follow-up methods, baseline characteristics and the extent of CVAE reporting within the published article were recorded. Data were extracted from primary manuscripts, supplementary appendices and publicly accessible trial protocols. Trial registration numbers were used to search platforms to identify additional protocol data where required.

### CV adverse events

CVAEs were defined as any AE recorded as a cardiac disorder under the Common Terminology Criteria for Adverse Events (CTCAE) which grades severity from 1 to 5, where grade 1 ‘mild’, grade 2 ‘moderate’, grade 3 ‘severe but not immediately life-threatening’, grade 4 ‘life-threatening’ and grade 5 ‘fatal event’. Myocardial infarction (MI) and acute coronary syndrome (ACS) were reported together under the ‘myocardial infarction’ category. AEs not classified as cardiac by CTCAE but meeting prespecified CV or stroke endpoints, based on Food and Drug Administration (FDA) endorsed Hicks’ criteria (eg, sudden death), were also considered CVAEs.^15^

## Results

**Figure 1:**
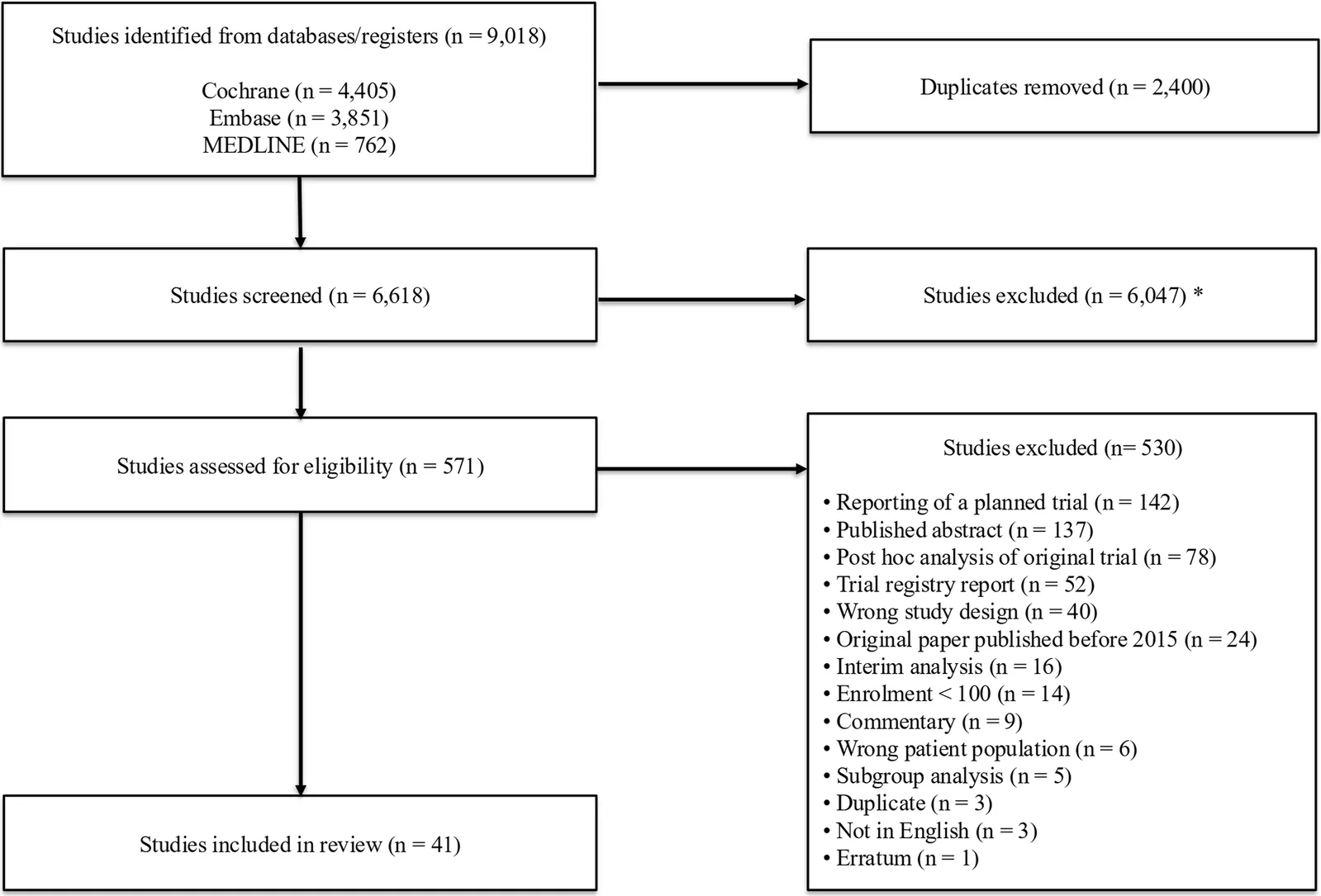
Preferred Reporting Items for Systematic Reviews and Meta-Analyses (PRISMA) diagram. PRISMA diagram illustrating the identification and selection of phase III randomised multiple myeloma (MM) trials included in this systematic review. Databases were searched for studies published between 2015 and 2025. After removal of duplicates and screening of titles, abstracts and full texts, 41 trials enrolling 20 144 participants met eligibility criteria. *Records were excluded at the title and abstract screening stage where they did not meet the predefined PICO eligibility criteria (online supplemental table 1). Most commonly, they were not phase III randomised controlled trials of an MM drug therapy.

The search identified 9018 references which were screened (figure 1). The final analysis included 41 phase III randomised drug trials with a total of 20 144 participants, published between 2015 and 2025 (table 1). Across these contemporary MM drug trials, study interventions were dominated by monoclonal antibodies (15 trials, 37%) and proteasome inhibitors (11 trials, 27%). The remaining 15 trials investigated targeted therapies (5 trials, 12%), immunomodulating agents (5 trials, 12%), alkylating agents (2 trials, 5%) and other novel therapies (3 trials, 7%). Corticosteroids were administered in 39 trials (95%) in combination with myeloma-specific therapies according to regimen schedules.

**Table 1:** Amyloidosis exclusions in phase III multiple myeloma drug trials

First author (year)	N	Intervention	Comparator	Amyloidosis excluded	Amyloidosis exclusion criteria	Active amyloidosis screening	Baseline CV Investigations
Monoclonal antibodies							
Attal (2019)^32^	307	Isatuximab+pomalidomide+dexamethasone	Pomalidomide+dexamethasone	Yes	‘Active primary amyloid-light (AL) amyloidosis (evidence of end organ damage or receiving treatment for amyloidosis)’	–	Yes*
Dimopoulos (2016)^33^	569	Daratumumab+lenalidomide+dexamethasone	Lenalidomide+dexamethasone	Yes	‘Amyloidosis’	–	Yes*
Dimopoulos (2021)^34^	304	Daratumumab+pomalidomide+dexamethasone	Pomalidomide+dexamethasone	Yes	‘Amyloidosis’	–	Yes*
Dimopoulos (2020)^35^	466	Daratumumab+carfilzomib+dexamethasone	Carfilzomib+dexamethasone	Yes	‘Primary amyloidosis’ (asymptomatic disease accepted)	–	Yes*†‡
Facon (2024)^36^	446	Isatuximab+bortezomib+lenalidomide+dexamethasone	Bortezomib+lenalidomide+dexamethasone	Yes	‘Primary amyloidosis’	–	Yes*‡
Facon (2019)^37^	737	Daratumumab+lenalidomide+dexamethasone	Lenalidomide+dexamethasone	Yes	‘Primary amyloidosis’	–	Yes*
Fu (2023)^38^	220	Daratumumab+bortezomib+melphalan+prednisolone	Bortezomib+melphalan+prednisolone	Yes	‘Primary amyloidosis’	–	Yes*
Goldschmidt (2022)^39^	660	Isatuximab+bortezomib+lenalidomide+dexamethasone	Bortezomib+lenalidomide+dexamethasone	Yes	‘Systemic AL amyloidosis’ (except for AL amyloidosis of the skin or the bone marrow)	–	Yes*‡
Mateos (2018)^40^	706	Daratumumab+bortezomib+melphalan+prednisolone	Bortezomib+melphalan+prednisolone	Yes	‘Primary amyloidosis’	–	Yes*
Mateos (2020)^41^	522	Subcutaneous daratumumab	Intravenous daratumumab	Yes	‘Amyloidosis’	–	Yes*
Moreau (2021)^42^	302	Isatuximab+carfilzomib+dexamethasone	Carfilzomib+dexamethasone	Yes	‘Known amyloidosis’	–	Yes*‡
Palumbo (2016)^43^	498	Daratumumab+bortezomib+dexamethasone	Bortezomib+dexamethasone	Yes	‘Amyloidosis’	–	Yes*
Usmani (2025)^44^	395	Daratumumab+lenalidomide+dexamethasone	Bortezomib+lenalidomide+dexamethasone	No	–	–	Yes*
Dimopoulos (2022)^17^	742	Elotuzumab+lenalidomide+dexamethasone	Lenalidomide+dexamethasone	Yes	‘Known or suspected cardiac amyloidosis’	–	Yes*‡
Lonial (2015)^18^	646	Elotuzumab+lenalidomide+dexamethasone	Lenalidomide+dexamethasone	Yes	‘Known or suspected cardiac amyloidosis’	–	Yes*‡
Proteasome inhibitors							
Dimopoulos (2024)^45^	454	Once weekly Carfilzomib+lenalidomide +dexamethasone	Twice weekly Carfilzomib+lenalidomide +dexamethasone	Yes	‘Symptomatic amyloidosis’	–	Yes*
Dimopoulos (2016)^46^	929	Carfilzomib+dexamethasone	Bortezomib+dexamethasone	Yes	‘Amyloidosis’	–	Yes*
Dimopoulos (2020)^47^	706	Ixazomib	Placebo	Yes	‘Primary amyloidosis’	–	Yes*
Durie (2017)^48^	525	Bortezomib+lenalidomide+dexamethasone	Lenalidomide+dexamethasone	No	–	–	Yes*
Facon (2019)^49^	955	Carfilzomib+melphalan+prednisolone	Bortezomib+melphalan+prednisolone	Yes	‘Amyloidosis’	–	No
Hajek (2016)^50^	315	Carfilzomib	Corticosteroid±cyclophosphamide	No	–	–	No
Kumar (2020)^51^	913	Carflizomib+lenalidomide+dexamethasone	Bortezomib+lenalidomide +dexamethasone	No	–	–	No
Leleu (2024)^52^	270	Isatuximab+bortezomib+lenalidomide+dexamethasone	Isatuximab+lenalidomide +dexamethasone	Yes	‘Primary amyloidosis’	–	No
Montefusco (2020)^53^	155	Bortezomib+cyclophosphamide+dexamethasone	Lenalidomide+cyclophosphamide +dexamethasone	No	–	–	No
Moreau (2016)^54^	722	Ixazomib+lenalidomide+dexamethasone	Lenalidomide+dexamethasone	Yes	‘Primary amyloidosis’	–	Yes*
Stewart (2015)^55^	792	Carfilzomib+lenalidomide+dexamethasone	Lenalidomide+dexamethasone	No	–	–	Yes*
Immuno-modulators							
Stewart (2015)^56^	306	Melphalan+prednisolone+thalidomide	Melphalan+prednisolone+lenalidomide	No	–	–	No
Magarotto (2016)^57^	654	Melphalan+prednisolone+lenalidomide or cyclophosphamide+prednisolone+lenalidomide	Dexamethasone+lenalidomide	No	–	–	No
Zweegman (2016)^58^	637	Melphalan+prednisolone+lenalidomide	Melphalan+prednisolone+thalidomide	Yes	‘Systemic light chain amyloidosis’	–	No
Larocca (2021)^59^	210	Lenalidomide+dexamethasone followed by maintenance lenalidomide	Continuous lenalidomide+dexamethasone	No	–	–	No
Richardson (2019)^60^	559	Bortezomib+dexamethasone+pomalidomide	Bortezomib+dexamethasone	No	–	–	Yes*
Targeted therapies							
Dimopoulos (2024)^61^	302	Belantamab mafodotin+pomalidomide+dexamethasone	Pomalidomide+bortezomib +dexamethasone	Yes	‘Symptomatic amyloidosis’	–	Yes*
Dimopoulos (2023)^62^	325	Belantamab mafodotin	Pomalidomide+dexamethasone	Yes	‘Primary amyloidosis’ (asymptomatic disease accepted)	–	Yes*†‡
Grosicki (2020)^63^	402	Once weekly selinexor+bortezomib+dexamethasone	Twice weekly bortezomib +dexamethasone	Yes	‘Systemic AL amyloid’	–	Yes*
Hungria (2024)^64^	494	Belantamab mafodotin+bortezomib+dexamethasone	Daratumumab+bortezomib +dexamethasone	Yes	‘Symptomatic amyloidosis’	–	Yes*
Kumar (2020)^65^	291	Venetoclax+bortezomib+dexamethasone	Bortezomib+dexamethasone	Yes	‘Amyloidosis’	–	Yes*
Alkylating agents							
Niesvizky (2015)^27^	502	Bortezomib+thalidomide+dexamethasone or bortezomib+melphalan+prednisolone	Bortezomib+dexamethasone	No	–	–	No
Schjesvold (2022)^66^	495	Melphalan+dexamethasone	Pomalidomide+dexamethasone	Yes	‘Concurrent symptomatic amyloidosis’	–	Yes*
Other agents							
Oriol (2024)^67^	170	Nivolumab+pomalidomide+dexamethasone	Pomalidomide+dexamethasone	Yes	‘Primary amyloidosis without active myeloma’	–	Yes*
Puig (2019)^68^	286	Clarithromycin+lenalidomide+dexamethasone	Lenalidomide+dexamethasone	Yes	‘Symptomatic primary amyloidosis’	–	No
Spicka (2019)^19^	255	Plitidepsin+dexamethasone	Dexamethasone	Yes	‘Cardiac amyloidosis’	–	Yes*†‡

* ECG.

† Cardiac biomarkers.

‡ CV imaging.

CV, cardiovascular.

### Trial eligibility criteria and screening

#### Amyloidosis

30 trials (14 818 participants, 74%) had amyloidosis exclusion criteria (table 1). Exclusion varied by treatment class, being more frequent in monoclonal antibody (14/15, 93%) and targeted therapy (5/5, 100%) trials than in proteasome inhibitor (6/11, 55%) or immunomodulator (1/5, 20%) trials. The terminology used for these exclusions was variable. The most common terms were: ‘amyloidosis’, ‘primary amyloidosis’ and ‘symptomatic amyloidosis’. Three trials specifically allowed the inclusion of participants with asymptomatic amyloidosis. Furthermore, three trials specifically excluded patients with ‘cardiac amyloidosis’ rather than systemic amyloidosis. In the manuscripts and protocols relating to these trials, none detailed how diagnoses of amyloidosis were ascertained.

No trial reported active screening specifically for amyloidosis. One trial (637 participants, 3%) provided reasons for screen failure related to amyloid disease. Notably, this trial did not include any CV investigations as part of the screening or baseline assessment yet excluded participants with cardiac amyloidosis.

#### Non-amyloid CVD

40 trials (19 749 participants, 98%) had exclusion criteria relating to CVD. However, these varied widely and lacked uniform definitions (table 2). The most common exclusion criteria were prior HF, MI/unstable angina, hypertension and arrhythmia, and these domains were applied across all treatment classes. Both the definitions and eligibility thresholds varied widely across studies, often relying on subjective, non-standardised descriptors such as ‘uncontrolled’ or ‘clinically significant’, with inconsistent incorporation of objective measures, including left ventricular ejection fraction (LVEF) or prespecified blood pressure cut-offs. Several eligibility criteria demonstrated treatment class–specific patterns: blood pressure criteria were more frequent in proteasome inhibitor and targeted therapy trials and QTc-based criteria were largely confined to monoclonal antibody trials. Immunomodulator trials incorporated the least specific criteria, and venous thromboembolism was infrequently addressed. Where a corrected QT interval (QTc) eligibility criterion was applied, the correction formula was similarly inconsistently reported. Eight trials reported the use of Fridericia’s correction and three did not state a method.

**Table 2:** Non-amyloid cardiovascular exclusion criteria in phase III multiple myeloma drug trials

First author (year)	N	Heart failure and left ventricular dysfunction		Vascular				Blood pressure	Arrhythmia and QT	
		Symptoms/ NYHA	LV dysfunction/ LVEF	CAD	Peripheral arterial disease	Venous thromboembolism	Cerebrovascular disease		Arrhythmia	QTc interval
Monoclonal antibodies										
Attal (2019)^32^	307	–	Significant cardiac dysfunction	MI*, unstable angina, major surgery†	–	–	–	–	–	–
Dimopoulos (2016)^33^	569	≥III	–	MI‡, unstable angina	–	–	–	–	Cardiac arrhythmia CTCAE V4 grade ≥2 or clinically significant ECG abnormalities	>500 m/s^F^
Dimopoulos (2021)^34^	304	≥III	–	MI‡, unstable angina, major surgery†	–	–	–	–	Cardiac arrhythmia CTCAE V4 grade ≥3; Clinically significant ECG abnormalities	>470ms^F^
Dimopoulos (2020)^35^	466	≥III	<40%	MI§, symptomatic ischaemia, major surgery¶	–	–	–	>159/99	Uncontrolled; clinically significant ECG abnormalities	>470ms^NS^
Facon (2024)^36^	446	≥III	<40%	MI‡, unstable angina‡, CABG‡	Peripheral artery bypass‡	PE or uncontrolled thromboembolic event**	Stroke or TIA‡	Poorly controlled‡	Grade ≥3 arrhythmias‡; 2nd/3rd degree AV block‡	–
Facon (2019)^37^	737	≥III	–	MI*, unstable angina*, major surgery†	–	–	–	–	Cardiac arrhythmia CTCAE V4 grade ≥2 or clinically significant ECG abnormalities	>470ms^F^
Fu (2023)^38^	220	≥III		MI‡, unstable angina, major surgery†	–	–	–	–	Uncontrolled	>470 ms^0^
Goldschmidt (2022)^39^	660	≥III	<40%	–	–	–	–	–	–	-
Mateos (2018)^40^	706	≥III	–	MI*, unstable angina, major surgery†	–	–	–	–	Cardiac arrhythmia CTCAE V4 grade ≥2 or clinically significant ECG abnormalities	>470 m/s^F^
Mateos (2020)^41^	522	≥III	–	MI‡, uncontrolled angina, major surgery†	–	–	–	–	Cardiac arrhythmia CTCAE V4 grade ≥2 or clinically significant ECG abnormalities	>470 m/s^F^
Moreau (2021)^42^	302	≥III‡	<40%	MI‡, unstable angina‡, CABG‡	Peripheral artery bypass‡	PE or uncontrolled thromboembolic event**	Stroke or TIA‡	–	Grade ≥3 arrhythmia	–
Palumbo (2016)^43^	498	≥III	–	MI‡, unstable angina, major surgery†	–	–	–	–	Cardiac arrhythmia CTCAE V4 grade ≥2 or clinically significant ECG abnormalities	>470 m/s^F^
Usmani (2025)^44^	395	–	–	–	–	–	–	–	–	–
Dimopoulos (2022)^45^	742	≥III	–	MI‡, uncontrolled angina, major surgery¶	–	–	Prior cerebrovascular event with persistent neurologic deficit	Uncontrolled	Uncontrolled	–
Lonial (2015)^18^	646	≥III	–	MI‡, uncontrolled angina, major surgery¶	–	–	Cerebrovascular event with persistent neurology	Uncontrolled	Uncontrolled	–
Proteasome inhibitors										
Dimopoulos (2024)^45^	454	≥III	<40%	MI§, symptomatic ischaemia§, major surgery¶	–	–	–	≥160/100	Uncontrolled	>470 m/s^NS^
Dimopoulos (2016)^46^	929	≥III	<40%	MI§, symptomatic ischaemia, major surgery¶	–	–	–	–	Conduction abnormalities uncontrolled by conventional intervention	–
Dimopoulos (2020)^47^	706	Uncontrolled congestive heart failure‡	–	MI‡; unstable angina‡; major surgery†	–	–	–	Uncontrolled	Uncontrolled‡	
Durie (2017)^48^	525	≥III	–	MI‡	–	–	History of cerebral vascular accident with persistent neurologic deficit	Poorly controlled	History of treatment for clinically significant ventricular cardiac arrhythmias	–
Facon (2019)^49^	955	≥III	<40%	MI‡, symptomatic ischaemia‡,	–	–	–	–	Conduction abnormalities uncontrolled by conventional intervention	–
Hajek (2016)^50^	315	≥III	–	MI**, symptomatic cardiac ischaemia, major surgery††	–	–	–	–	Conduction system abnormalities uncontrolled by conventional intervention	–
Kumar (2020)^51^	913	≥III‡		MI‡, unstable angina	–	–	–	Uncontrolled	Uncontrolled	–
Leleu (2024)^52^	270	≥III	<40%	MI‡, unstable angina, major surgery†	–	–	–	–	Uncontrolled, CTCAE V5 grade ≥2 or clinically significant ECG abnormalities	–
Montefusco (2020)^53^	155	–	<45%	MI*	–	PE**	–	–	–	–
Moreau (2016)^54^	722	Symptomatic‡	–	MI‡, unstable angina‡, major surgery†	–	–	–	Uncontrolled	Uncontrolled‡	–
Stewart (2015)^55^	792	≥III	–	MI§, uncontrolled angina, severe CAD, major surgery††	–	–	–	Uncontrolled	Severe uncontrolled ventricular arrhythmias, sick sinus syndrome or grade 3 conduction system abnormalities unless subject has a pacemaker	–
Immuno-modulators										
Stewart (2015)^56^	306	Symptomatic	–	Unstable angina	–	–	–	Uncontrolled	Uncontrolled	–
Magarotto (2016)^57^	654	Uncontrolled or severe cardiovascular disease. No further definitions provided from available study documents								
Zweegman (2016)^58^	637	≥II	–	–	–	–	–	–	–	–
Larocca (2021)^59^	210	Any significant medical disease or conditions that, in the investigator’s opinion, may interfere with protocol adherence or subject’s ability to give informed consent or could place the subject at unacceptable risk.								
Richardson (2019)^60^	559	≥III	–	MI*, unstable angina, major surgery†	–	–	–	–	Clinically significant abnormal ECG	–
Targeted therapies										
Dimopoulos (2024)^61^	302	≥III	–	MI**, ACS**, unstable angina**, coronary angioplasty**, stenting** or bypass grafting**	Arterial Thromboembolism**	Venous thromboembolism**	–	Uncontrolled	Clinically significant untreated arrhythmias or ECG abnormalities (Mobitz 2nd or 3rd degree AV block)	–
Dimopoulos (2023)^62^	325	≥III	–	MI**, ACS**, unstable angina**, coronary angioplasty**, stenting** or bypass grafting**	–	–	–	Uncontrolled	Clinically significant untreated arrhythmias or ECG abnormalities (Mobitz 2nd or 3rd degree AV block)	–
Grosicki (2020)^63^	402	≥III	<40%	MI**, symptomatic ischaemia	–	–	–	Uncontrolled	Uncontrolled clinically significant conduction abnormalities, Ventricular tachycardia, on antiarrhythmics	–
Hungria (2024)^64^	494	≥III	–	MI**, ACS**, unstable angina**, coronary angioplasty**, stenting** or bypass grafting**	–	–	–	Uncontrolled	Clinically significant untreated arrhythmias or ECG abnormalities (Mobitz 2nd or 3rd degree AV block)	–
Kumar (2020)^65^	291	≥III	–	MI‡, uncontrolled angina, major surgery§	–	–	–	Uncontrolled	Uncontrolled	–
Alkylating agents										
Niesvizky (2015)^27^	502	≥III	–	MI‡, unstable angina, major surgery¶	–	–	–	–	Uncontrolled ventricular arrhythmias or significant conduction system abnormalities	–
Schjesvold (2022)^66^	495	–	–	MI‡, major surgery¶	–	≥grade 3 thromboembolic event‡	–	Uncontrolled	Significant conduction system abnormalities	>470 m/s^F^
Other agents										
Oriol (2024)^67^	170	≥III	–	MI*, poorly controlled angina, major cardiac surgery‡‡	–	–	–	Uncontrolled	Serious arrhythmia	–
Puig (2019)^68^	286	≥III	<35%	MI‡, uncontrolled angina	–	Thromboembolic event¶	–	–	Severe uncontrolled ventricular arrhythmias or active conduction system abnormalities	–
Spicka (2019)^19^	255	Symptomatic*	Below lower limit of normal	MI*, presence of angina*	–	–	–	–	Symptomatic arrhythmia	CTCAE grade ≥2^F^

QT correction formula used by the trial.

* Within 12 months.

† Within 2 weeks.

‡ Within 6 months.

§ Within 4 months.

¶ Within 4 weeks.

** Within 3 months.

†† Within 3 weeks.

‡‡ Within 2 months.

ACS, acute coronary syndrome; AV, atrioventricular; CABG, coronary artery bypass graft; CAD, coronary artery disease; CTCAE, Common Terminology Criteria for Adverse Events; F, Fridericia; LV, left ventricle; LVEF, left ventricular ejection fraction; MI, myocardial infarction; NS, not stated; NYHA, New York Heart Association (classification); PE, pulmonary embolism; QTc, corrected QT interval; TIA, transient ischaemic attack.

### Baseline CV investigation

The use of CV investigations at baseline was very variable (online supplemental table 3 and online supplemental figure 1). A large proportion of trials (30 trials; 14 941 participants, 74%) incorporated CV investigations that would have the potential to identify features compatible with cardiac amyloidosis. This varied by treatment class, with at least one form of baseline CV investigation performed in all monoclonal antibody (n=15) and targeted therapy (n=5) trials, but in only 55% of proteasome inhibitor (n=11) and 20% of immunomodulator (n=5) trials. However, these investigations appeared to be performed for non-amyloid CV eligibility assessment rather than targeted amyloidosis detection (online supplemental figure 2).

Echocardiograms were performed in nine trials (4771 participants, 24%) at baseline. However, no trial reported baseline echocardiographic findings in either the main manuscript or supplementary material. Cardiac magnetic resonance (CMR) was used in one trial (446 participants, 2%). In this study, baseline CMR was performed ‘as required’ without further specification and the total number of scans performed and findings were not reported.

ECG was the most frequent baseline CV investigation and was included in 30 trials (14 941 participants, 74%). Only two trials described ECG interpretation criteria, classifying results as ‘normal’, ‘abnormal’ (clinically significant or not clinically significant) or ‘unable to evaluate’. One of these reported baseline ECG findings, categorising them as ‘normal’ or ‘abnormal, not clinically significant’.

Three trials (1046 participants, 5%) included baseline assessment of cardiac biomarkers.

### Reporting of baseline characteristics

#### Cardiac amyloidosis

11 trials (5326 participants, 26%) permitted enrolment of patients with cardiac amyloidosis. However, none of these trials reported the number of patients with amyloidosis enrolled.

#### Non-amyloid CVD

Baseline CV characteristics were reported in only one trial (502 participants, 2%), in which previous MI, HF, cerebrovascular disease, peripheral vascular disease and diabetes were described.

### Adverse event reporting

#### Cardiac amyloidosis

Three trials reported incident amyloidosis in the Supplementary Materials as a serious adverse outcome, with all three specifying cardiac amyloidosis. In addition, four trials listed serious adverse events with features suggestive of amyloidosis, including ‘septal thickening with atrioventricular block’, ‘cardiac hypertrophy’ and ‘restrictive cardiomyopathy’. However, in these reports no further clinical or pathological details were provided, limiting the ability to confirm the diagnosis of amyloidosis. No trial separately analysed the outcomes of the patients diagnosed with amyloidosis.

#### Non-amyloid CVD

**Figure 2:**
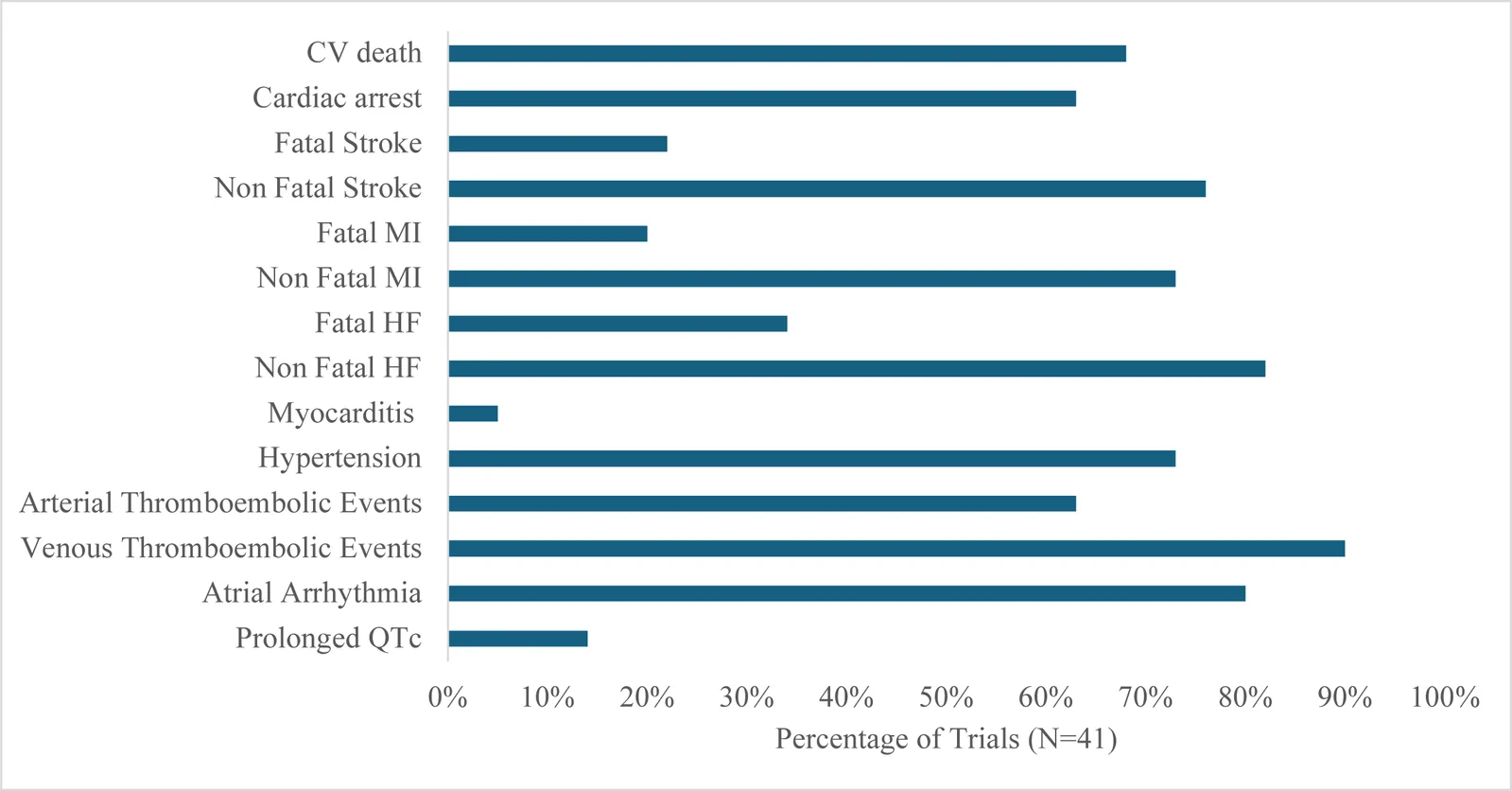
Percentage of trials reporting cardiovascular (CV) adverse events. Reporting varied across outcomes including myocardial infarction (MI), heart failure (HF), stroke, atrial arrhythmia, hypertension and thromboembolic events. Events were investigator-reported and definitions varied between trials, limiting cross-study comparisons and interpretation of CV safety outcomes. QTc, corrected QT interval.

All 41 trials reported adverse events using CTCAE definitions and severity grading. None of the trials employed the FDA-endorsed, standardised Hicks’ criteria for reporting CV events.^15^ Substantial variability was observed in the terminology used both within individual trials and across the studies reviewed. One trial reported adverse events as ‘CV disorders’ and ‘neurological disorders’ without offering any further granularity. In trials that did not report CVAEs, it remained unclear whether this reflected a true absence of such events or incomplete reporting. AEs were reported by site investigators, and no trial had a specialist CV event adjudication panel (figure 2).

#### CV death

CV death was reported in 28 trials (14 112 participants, 70%). Three trials (1210 participants, 6%) did not report the mode of AE death and recorded it as ‘cardiology disorder’ or ‘cardiology toxicity’. No trial reported the total number of CV deaths.

#### Myocardial infarction

MI was reported in 32 trials (16 830 participants, 84%). Two trials only reported fatal MI. 21 trials (11 589 participants, 58%) used multiple definitions for MI adverse event reporting.

#### HF and left ventricular systolic dysfunction

HF was reported in 35 trials (18 181 participants, 90%). One trial only reported fatal cases. Incident left ventricular systolic dysfunction (LVSD) was reported in 12 trials (6446 participants, 32%) and 3 of these included protocol-defined serial echocardiography. Among studies that reported HF and LVSD events, 13 different definitions for HF and/or LVSD were identified with many trials using several definitions within the same manuscript. The mean number of definitions per study was 3, with 1 trial including 7 definitions.

#### Stroke

Stroke was reported in 32 trials (16 654 participants, 82%). One trial only reported fatal stroke. Trials reporting non-fatal strokes used several definitions. 19 trials (9329 participants, 46%) employed at least one additional term beyond ischaemic and haemorrhagic stroke. These included ‘cerebrovascular accident’, ‘embolic cerebral infarction’ and ‘cerebral infarction’.

#### Venous and peripheral arterial thromboembolic events

Venous thrombotic events were reported in 37 trials (18 867 participants, 94%). 26 trials reported arterial thrombotic events (13 832 participants, 69%). Three trials (1348 participants) reported venous thromboembolic deaths, and two trials (1236 participants) reported death related to arterial thrombotic events. Two trials (1199 participants) reported death to unspecified thromboembolic events.

#### Hypertension

Hypertension was reported in 30 trials (14 286 participants, 71%).

#### Cardiac arrest and arrhythmia

Cardiac arrest was reported in 26 trials (13 791 participants, 68%). In 8 of these trials (4521 participants), ‘cardiac arrest’ and ‘cardiorespiratory arrest’ were reported as separate outcomes. One trial (913 participants) provided detail of the cardiac rhythm associated with the cardiac arrest.

Arrhythmias were reported in 35 trials (17 624 participants, 87%). The most frequently described were atrial fibrillation and supraventricular tachycardia. 14 trials (8771 participants, 44%) reported ‘sinus bradycardia’, ‘sinus tachycardia’ and ‘tachycardia’ as distinct adverse events in addition to standard arrhythmia classifications. Six trials (2799 participants, 14%) reported a prolonged QT interval. Three trials (1449 participants) reported arrhythmia without further specification.

#### Reporting by treatment class

The completeness of CVAE reporting varied across treatment classes. Reporting was most comprehensive in monoclonal antibody trials. Among immunomodulatory drug trials, venous thromboembolism was reported in all five trials, whereas hypertension and peripheral arterial disease were each reported in only two.

## Discussion

**Figure 3:**
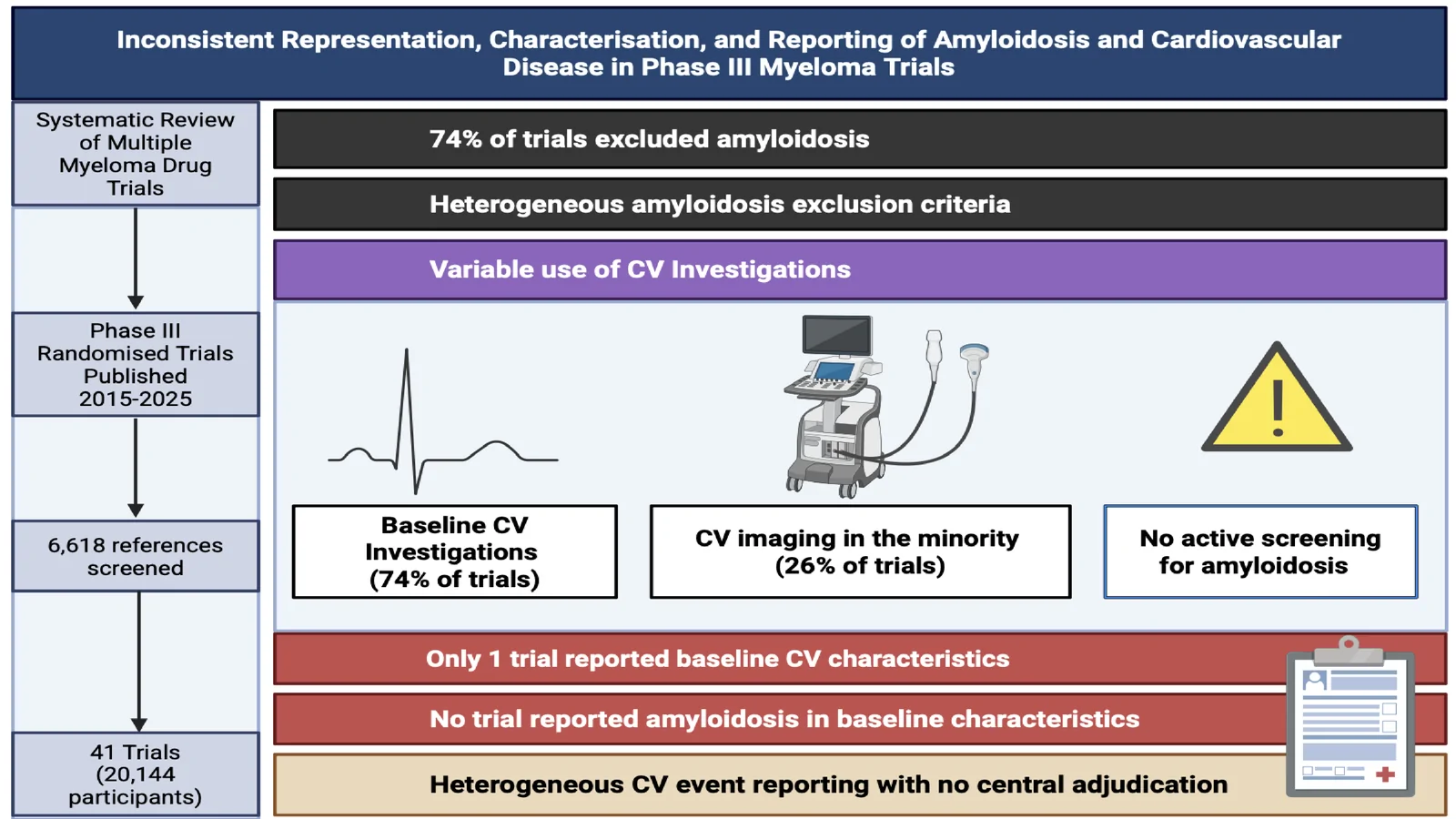
Amyloidosis and cardiovascular (CV) disease in phase III MM trials. Among 41 phase III MM trials (2015–2025), amyloidosis was frequently excluded (74%) using heterogeneous criteria, without systematic screening or assessment of cardiac involvement. Baseline CV investigations were variably applied and rarely reported, with minimal characterisation of underlying disease. CV adverse events were inconsistently defined, investigator-reported and not centrally adjudicated. These limitations hinder accurate interpretation of CV risk and reduce the generalisability of trial findings. MM, multiple myeloma.

In this systematic review of randomised phase III MM trial design and reporting, we identified substantial heterogeneity in how amyloidosis and CVD are incorporated into eligibility criteria, CV assessment and adverse event reporting. The key findings of this review are summarised in figure 3. Current phase III MM trials lack standardised approaches to CV and amyloidosis phenotyping, limiting the comparability of CV data across trials.

Although CV trial design varied across studies, one of our most relevant findings relates to cardiac amyloidosis, which is a dominant prognostic factor in patients with MM.^16^ While cardiac amyloidosis is a frequent exclusion criterion in phase III MM trials, the extent to which this was characterised and reported at each stage of trial screening, enrolment and follow-up was limited and presents an opportunity for improvement in future trials.

Of the 30 studies that excluded amyloidosis, 27 applied non-specific exclusions referring to amyloidosis or systemic AL amyloidosis, whereas only a small minority (three trials) explicitly excluded cardiac amyloidosis, despite cardiac involvement being the dominant determinant of prognosis.

Protocol-defined screening approaches for the identification of cardiac amyloidosis were not used and exclusion definitions varied widely. Most trial protocols did not specify diagnostic criteria, biomarker thresholds or imaging requirements. Notably, among the three trials^17–19^ that specifically excluded cardiac amyloidosis rather than systemic amyloidosis, only one mandated the baseline assessment of cardiac biomarkers, in addition to ECG and cardiac imaging. Only 5% of the 20 144 patients included in myeloma trials over the past 10 years had cardiac biomarker measurement at baseline, a key diagnostic for suspicion of amyloidosis or HF. CV investigations were limited and inconsistently applied. Assessments largely focused on arrhythmia identification and assessment of QTc and left ventricular systolic function, with little integration between modalities. This approach may have limited sensitivity for detection of cardiac amyloidosis, which often presents with preserved ejection fraction and subtle electrocardiographic or biomarker abnormalities.^20 21^ The prevalence of clinically significant concurrent cardiac amyloidosis within broad MM trial populations remains uncertain. In the absence of structured screening or reporting, it is not possible to determine whether under-recognition of occult cardiac amyloidosis influenced efficacy outcomes or treatment-related CV safety signals. This remains a hypothesis requiring further evaluation rather than a demonstrated effect. We therefore interpret these findings as reflecting uncertainty in baseline characterisation and event attribution rather than proven clinical misclassification. Whether more structured CV and amyloidosis phenotyping would improve risk estimation warrants prospective evaluation.

In contrast to the trials reviewed here, which enrolled broad MM populations, the phase III ANDROMEDA trial^22^ specifically recruited patients with AL amyloidosis. The addition of daratumumab improved haematologic outcomes and survival free from major organ deterioration, providing important evidence for a clinically complex population for whom trial data are otherwise limited. ANDROMEDA incorporated structured eligibility criteria, systematic organ phenotyping and prespecified cardiac endpoints alongside haematologic outcomes. Adoption of similar design principles in MM trials may provide a useful model for improving baseline CV characterisation and facilitating more consistent CV assessment in future MM trials. A structured approach to CV and amyloidosis phenotyping using a pragmatic, tiered screening strategy might be considered for use in future MM trials. Baseline cardiac biomarkers (B-type natriuretic peptide (BNP) or N-terminal proBNP and high-sensitivity troponin), together with a standard 12 lead ECG, could provide a simple, low-cost initial screening step. Given the high prevalence of CVD in patients with MM, more consistent baseline echocardiography may also be considered to improve CV phenotyping and identify features that could raise suspicion for cardiac amyloidosis. More advanced investigations, including cardiac MRI or tissue biopsy could then be reserved for participants with abnormal findings, guided by specialist clinical assessment. This tiered approach could concentrate resource-intensive investigations on higher risk participants simultaneously to improve clinical care while remaining compatible with the practical constraints of multicentre clinical trials.

Non-amyloid CVD and CV risk factors are highly prevalent in MM^23^ and influence clinical decision-making and outcomes. Despite this, attention to harmonised eligibility criteria, baseline CV characteristics and CVAE reporting was limited.

We identified substantial variation in CVD eligibility criteria, often relying on subjective descriptors and non-standardised thresholds for HF, arrhythmias and vascular disease. In addition, QTc correction formula was inconsistently reported. This aligns with prior cardio-oncology evaluations showing that inconsistent criteria and definitions challenge interpretation and external validity.^13 24^ A proportion of this heterogeneity may reflect rational, drug-specific trial design rather than an absence of standardisation alone. Blood pressure criteria were more prevalent in proteasome inhibitor and targeted therapy trials, concordant with the established association between carfilzomib and hypertension. Other patterns, however, did not correspond to recognised class toxicities: QTc-based criteria were largely confined to monoclonal antibody trials despite QTc prolongation not being an established effect of these agents, possibly implying inherited or non-specific protocol design rather than drug-directed rationale. A comparable disconnect was evident in reporting.

Although trial exclusion based on pre-existing CVD may be justified for safety, inconsistent criteria and reporting limits extrapolation of trial results to ‘real-world’ patients with MM in whom CVD is common.^25^ Regulatory agencies and cardio-oncology societies have emphasised avoiding restrictive CV eligibility criteria.^26^

Despite the clinical relevance of CVD in MM, baseline CV comorbidities were reported in only one trial.^27^ In that study, HF, prior MI and cerebrovascular disease were uncommon (<10%). In contrast, population-based studies of newly diagnosed MM report much higher baseline rates of HF (33%), ischaemic heart disease (51%) and arrhythmias (51%) in a US SEER Medicare analysis,^28^ with a Finnish registry corroborating a high baseline CVD prevalence (27.9% rising to ~55% at 2 years).^25^ This highlights the difficulty in extrapolating trial results to typical clinical populations with CV comorbidity.^29^

CVAEs were frequently reported, indicating that CV safety surveillance was incorporated into most included studies. However, inconsistencies in definitions, terminology and reporting practices limited cross-trial comparability. Non-specific or outdated CV terminology was common, particularly for HF, with up to seven definitions for HF and LVSD identified in a single trial. Combined with reliance on investigator-reported events without independent adjudication, this increases the risk of misclassification and over-reporting or under-reporting. In addition, as observed in prior cardio-oncology trial evaluations,^13^ CVAE reporting often used frequency threshold-based criteria. This means that infrequent but clinically meaningful events are at risk of under-reporting and further diminishing the ability to perform meta-analysis. Collectively, the principal limitation was not the absence of CV safety data, but substantial heterogeneity in endpoint definitions, reporting thresholds and adjudication practices, limiting cross-trial comparability and pooled CV risk assessment.

Ongoing standardisation initiatives provide a clear path towards more reliable CVAE reporting. Adoption of CTCAE v6.0,^30^ together with harmonised CV toxicity definitions and grading through International Cardio-Oncology Society consensus recommendations,^31^ could improve consistency across oncology trials. Coupled with independent CV adjudication, these frameworks may enhance the accuracy, comparability and interpretability of CVAE outcomes in future trials.

### Limitations

Several limitations should be acknowledged. Pre-enrolment safety evaluations were not analysed, and longer-term follow-up reports were outside the scope of this review, limiting assessment of delayed CV and amyloidosis related complications. Stem cell transplantation and CAR T-cell therapy trials were not included; although these populations are often younger and have a lower burden of comorbidities, CV complications remain clinically relevant and warrant dedicated evaluation in future analyses. Restriction to phase III trials enrolling ≥100 participants and published from 2015 onwards was intentional in order to focus the review on recent practice-changing trials. Earlier-phase and smaller trials may include more intensive CV phenotyping that is not captured here. Eligibility was restricted to English-language publications, risking language bias. Our search covered three databases (MEDLINE, Embase and the Cochrane Library). While there is a small possibility that relevant trials may not have been captured within these criteria, we believe that our approach allowed the capture of the most relevant and representative, potentially practice-changing myeloma trials. Finally, this review relied on published manuscripts, supplementary appendices and publicly available protocols. Thus, absence of reported CV or amyloidosis assessments does not necessarily mean these evaluations were not performed in practice.

## Conclusions

This review of practice-changing phase III MM trials demonstrates substantial heterogeneity in the reporting and handling of AL amyloidosis and CVD across eligibility criteria, baseline characterisation and outcome reporting. AL amyloidosis was commonly used as an exclusion criterion without standardised screening or clear distinction between systemic and cardiac involvement, while non-amyloid CV comorbidities were inconsistently defined and assessed. Variability in CV eligibility criteria, baseline phenotyping and adverse-event reporting is consistent with the absence of a unified cardio-oncology trial framework.

These findings reflect uncertainty in baseline CV characterisation and CV event attribution rather than demonstrating patient-level misclassification or differences in clinical outcomes. Standardisation of CV eligibility criteria, baseline phenotyping and CV outcome reporting in future MM trials could improve comparability across studies and strengthen the evidence base for the use of increasingly effective myeloma therapies in patients with CV comorbidity frequently encountered in clinical practice.

## Supplementary material

10.1136/openhrt-2026-004240 online supplemental file 1

## Data Availability

All data relevant to the study are included in the article or uploaded as supplementary information.
