# Supplementary material for: Cardiovascular disease and amyloidosis in phase III multiple myeloma trials: a systematic review of eligibility, screening and event reporting
Source: Open Heart. 2026 Sep 1;13(2):e004240. doi: 10.1136/openhrt-2026-004240 (PMC13536086; doi:10.1136/openhrt-2026-004240)

Supplemental Material

Supplemental Table 1: PICO framework table.

Outcomes of interest using PICO framework and data points of interest

Population	Inclusion criteria:<br>- adult population (over 18 years old) with multiple myeloma<br>Exclusion criteria:<br>- animal studies.<br>- under 18
Intervention	Inclusion criteria:<br>- phase III trial<br>- randomised<br>- multiple myeloma drug trial<br>- published in English language<br>- published since 2015<br><br>The specific intervention exclusion criteria are listed below:<br>- Stem cell transplant<br>- Chimeric antigen receptor therapy<br>- population of treatment group of <100 patients,<br>-non-randomised trials<br>- meta-analysis,<br>- review articles or commentaries,<br>- subsequent therapy analysis,<br>- cost-effective analysis,<br>- published abstracts,<br>- patient reported outcomes,<br>- subgroup analysis,<br>- duplications,<br>- retrospective analysis.

Comparison	Eligible studies must compare intervention of interest against one or more of the following:<br><br>- Placebo<br><br>- Active controlled trials<br><br>- Open label studies
Outcome	Main outcomes<br><br>The primary outcomes include:<br><br>1. Identifying to what degree patients with cardiac AL amyloidosis are excluded from these clinical trials in the eligibility criteria.<br><br>2. Identifying to what extent patients with cardiac AL amyloidosis are enrolled in MM clinical trials<br><br>3. How cardiac AL amyloidosis are assessed and reported within these trials.<br><br>Measures of effect<br><br>This will be the proportion of clinical trials that exclude patients with AL amyloidosis, the proportion of trials including amyloidosis and reporting outcomes, the methods of amyloidosis screening in trial enrolment and whether outcomes are stratified by presence or absence of AL amyloidosis.<br><br>We will collect the following data points for the primary outcome :<br><br>Exclusion of AL amyloidosis:<br><br>1. Are AL amyloidosis related exclusions explicitly stated in the eligibility criteria<br><br>2. What diagnostic criteria were used for exclusion?<br><br>3. Were patients excluded with cardiac AL amyloidosis?<br><br>Representation of AL Amyloidosis in MM trials:<br><br>1. Are AL diagnoses reported in baseline characteristics or supplementary material?<br><br>2. What proportion of patients in MM trials have AL amyloidosis?<br><br>3. If patients with AL amyloidosis were included were their outcomes analysed separately<br><br>Screening for AL amyloidosis<br><br>1. Was AL Amyloidosis explicitly screened for before trial enrolment?<br><br>2. What screening methods were used?<br><br>3. Was troponin or NTproBNP measured?

	4. Was echocardiography used? Were any scoring methods implemented?<br><br>5. Was electrocardiography used? What parameters were looked at?<br><br>6. Was cardiac MRI used?<br><br>8. Was biopsy of any tissue performed? – including cardiac<br><br><br>Additional outcomes<br><br>Secondary outcome data points as follows:<br><br>Cardiovascular:<br><br>1. Were patients excluded with cardiovascular disease as defined by below:<br><br>- Hypertension<br><br>- Coronary artery disease (CAD)<br><br>- Heart failure<br><br>- Cardiomyopathy<br><br>- Cardiac amyloidosis<br><br>- Arrhythmia<br><br>- Valvular disease<br><br>- Arterial thromboembolic disease<br><br>- Venous thromboembolic disease<br><br>- Cerebrovascular disease<br><br>- Abnormal ECG<br><br>- Myocarditis<br><br>- Pericarditis<br><br>- Vasculitis<br><br>2. Are the diagnosis of cardiovascular disease of trial population available in baseline characteristics or supplementary materials?<br><br>3. Are adverse cardiovascular events reported?<br><br>4. How are adverse cardiovascular events defined?

Supplemental Table 2: Systematic review search terms

Database	Platform	Search strategy	Limits
MEDLINE	Ovid	1. exp MULTIPLE MYELOMA/ 2. myelom*.tw,kf. 3. exp Plasmacytoma/ 4. (plasm?cytom* or plasm?zytom* or plasma cytoma*).tw,kf. 5. (plasma* adj3 neoplas*).tw,kf. 6. (plasma cell adj1 (leukaem* or leukem* or tumor* or tumour*)).tw,kf. 7. ((plasmacytic* or plasmocytic* or plasmocyte*) adj1 (leukem* or leukaem*)).tw,kf. 8. kahler*.tw,kf. 9. 1 or 2 or 3 or 4 or 5 or 6 or 7 or 8 10. ("phase 3 clinical trial" or "phase III clinical trial").tw,kf. 11. (Phase 3 or Phase III).mp. 12. 10 or 11 13. 9 and 12	Records published from 2015 to date of extraction 2025
Embase	Ovid	1. multiple myeloma/ 2. myelom*.tw,kw. 3. plasmacytoma/ 4. plasma cell leukemia/ 5. (plasma* adj3 neoplas*).tw,kw. 6. (plasma cell adj1 (leukaem* or leukem* or tumor* or tumour* or neoplasm*)).tw,kw. 7. (plasm?cytom* or plasm?zytom* or plasma cytoma*).tw,kw. 8. ((plasmacytic* or plasmocytic* or plasmocyte*) adj1 (leukem* or leukaem*)).tw,kw. 9.	Records published from 2015 to date of extraction 2025

		kahler*.tw,kw. 10. 1 or 2 or 3 or 4 or 5 or 6 or 7 or 8 or 9 11. ("phase 3 clinical trial" or "phase III clinical trial").tw,kw. 12. (Phase 3 or Phase III).mp. 13. 11 or 12 14. 10 and 13	
CENTRAL	Cochrane Library	1. MeSH descriptor: [Multiple Myeloma] explode all trees 2. myelom*:ti,ab,kw 3. MeSH descriptor: [Plasmacytoma] explode all trees 4. (plasm*cytom* or plasm*zytom* or plasma cytoma*):ti,ab,kw 5. (plasma* NEAR/3 neoplas*):ti,ab,kw 6. (plasma cell NEAR/1 (leukaem* or leukem* or tumor* or tumour*)):ti,ab,kw 7. ((plasmacytic* or plasmocytic* or plasmocyte*) NEAR/1 (leukem* or leukaem*)):ti,ab,kw 8. kahler*:ti,ab,kw 9. #1 or #2 or #3 or #4 or #5 or #6 or #7 or #8 10. "Phase III" or "Phase 3" 11. #9 and #10	Records published from 2015 to date of extraction 2025

Supplemental Table 3. Cardiovascular investigations used in Phase III multiple myeloma drug trials

	Cardiac biomarkers		Electrocardiography		Cardiovascular Imaging *	
**First author (Year)**	Baseline	Longitudinal	Baseline	Longitudinal	Baseline	Longitudinal
Attal (2019)	-	-	Yes	(As clinically indicated)	-	-
Dimopoulos (2024)	-	-	Yes	(As clinically indicated)	-	-
Dimopoulos (2023)	NTproBNP	(As clinically indicated)	Yes	Yes	Yes	Yes
Dimopoulos (2024)	-	-	Yes	-	-	-
Dimopoulos (2016)	-	-	Yes	Yes	Yes	Yes
Dimopoulos (2016)	-	-	Yes	Yes	-	-
Dimopoulos (2022)	-	-	Yes	-	Yes	-
Dimopoulos (2020)	-	-	Yes	-	-	-
Dimopoulos (2021)	-	-	Yes	(End of treatment)	-	-
Dimopoulos (2020)	NTproBNP	(As clinically indicated)	Yes	Yes	Yes	Yes
Durie (2017)	-	-	Yes	-	-	-
Facon(2024)	-	-	Yes	(End of treatment)	Yes[b]	(End of treatment)
Facon (2019)	-	-	Yes	Yes	-	-
Facon (2019)	-	-	-	-	-	-
Fu (2023)	-	-	Yes	Yes	-	-
Goldschmidt (2022)	-	-	Yes	Yes	Yes	(End of treatment)
Grosicki (2020)	-	-	Yes	(End of treatment)	-	-
Hajek (2016)	-	-	-	-	-	-
Hungria (2024)	-	-	Yes	-	-	-
Kumar (2020)	-	-	Yes[a]	-	-	-
Kumar (2020)	-	-	-	-	-	-
Larocca (2021)	-	-	-	-	-	-
Leleu (2024)	-	-	-	-	-	-
Lonial (2015)	-	-	Yes	-	Yes	-
Magarotto (2016)	-	-	-	-	-	-
Mateos (2018)	-	-	Yes	Yes	-	-
Mateos(2020)	-	-	Yes	(End of treatment)	-	-
Montefusco (2020)	-	-	-	-	-	-
Moreau (2021)	-	-	Yes	Yes	Yes	-
Moreau (2016)	-	-	Yes	Yes	-	-
Niesvizky (2015)	-	-	-	-	-	-

Oriol (2024)	-	-	Yes	-	-	-
Palumbo (2016)	-	-	Yes	Yes	-	-
Puig (2019)	-	-	-	-	-	-
Richardson (2019)	-	-	Yes[a]	Yes[a]	-	-
Schjesvold (2022)	-	-	Yes	Yes	-	-
Spicka (2019)	Troponin I	Troponin I	Yes	-	Yes	-
Stewart (2015)	-	-	-	-	-	-
Stewart (2015)	-	-	Yes	-	-	-
Usmani et al (2025)	-	-	Yes	-	-	-
Zweegman et al (2016)	-	-	-	-	-	-

Key

*Echocardiography. Other imaging modalities in addition to echo highlighted in key below

a Documented as normal, abnormal (Not clinically significant or clinically significant) or unable to evaluate;

b - Cardiac MRI

Supplemental Figure Legend

Supplemental Figure 1: Baseline and longitudinal cardiovascular investigations in multiple myeloma trials

Overview of cardiovascular investigations incorporated into phase III MM trials at baseline and during follow-up. Electrocardiography was the most commonly reported assessment, while cardiac imaging and cardiac biomarkers were used less frequently. These investigations were primarily intended to assess cardiovascular eligibility rather than to identify cardiac amyloidosis.

Supplemental Figure 2: Amyloidosis exclusions in multiple myeloma trials

Amyloidosis exclusions and assessments in phase III MM trials. Many trials set out to exclude participants with amyloidosis but did not describe diagnostic criteria or perform systematic screening for cardiac involvement. Some studies included cardiovascular investigations without amyloid-specific evaluation, while others excluded amyloidosis without cardiovascular assessment.

Supplemental Figure 1

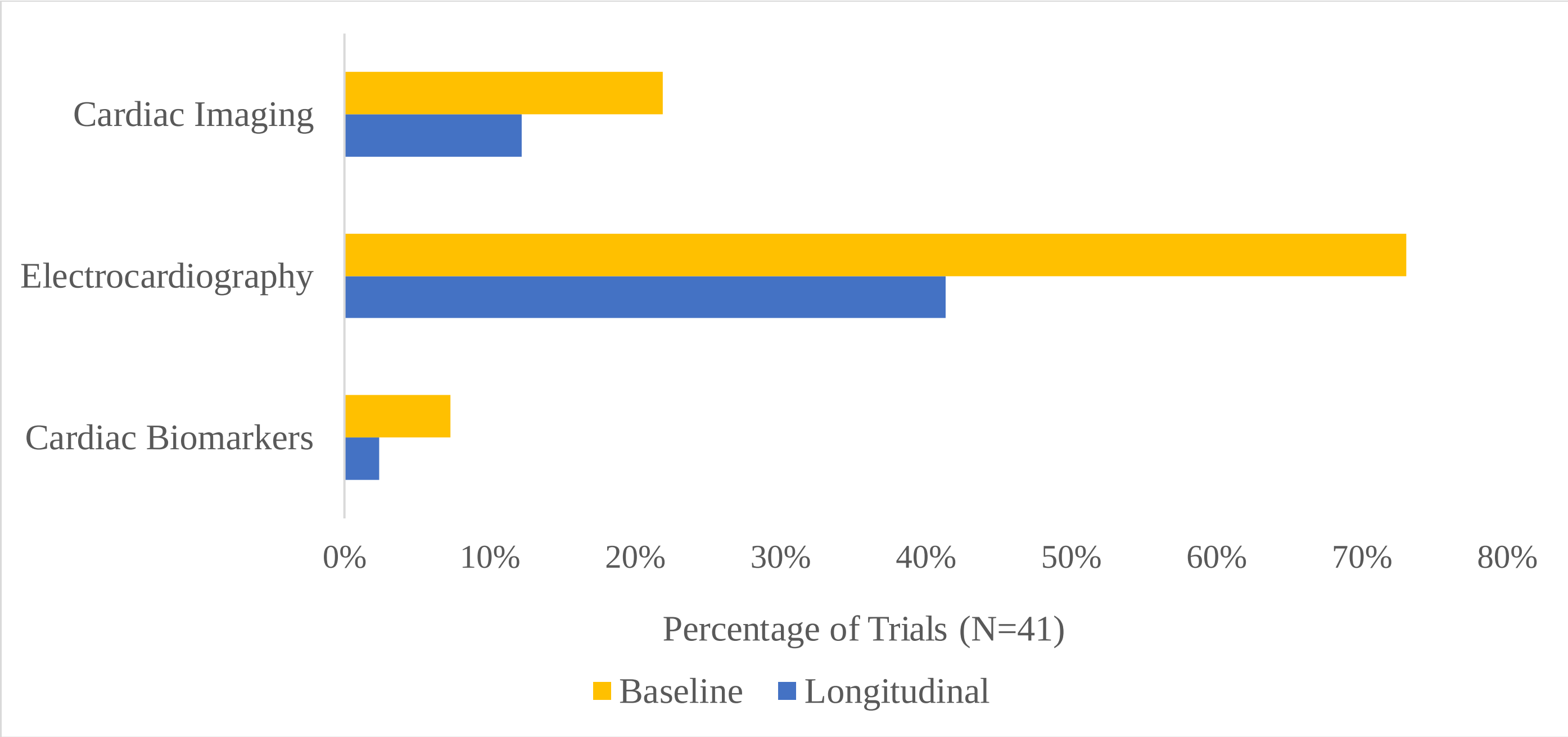

Supplemental Figure 2

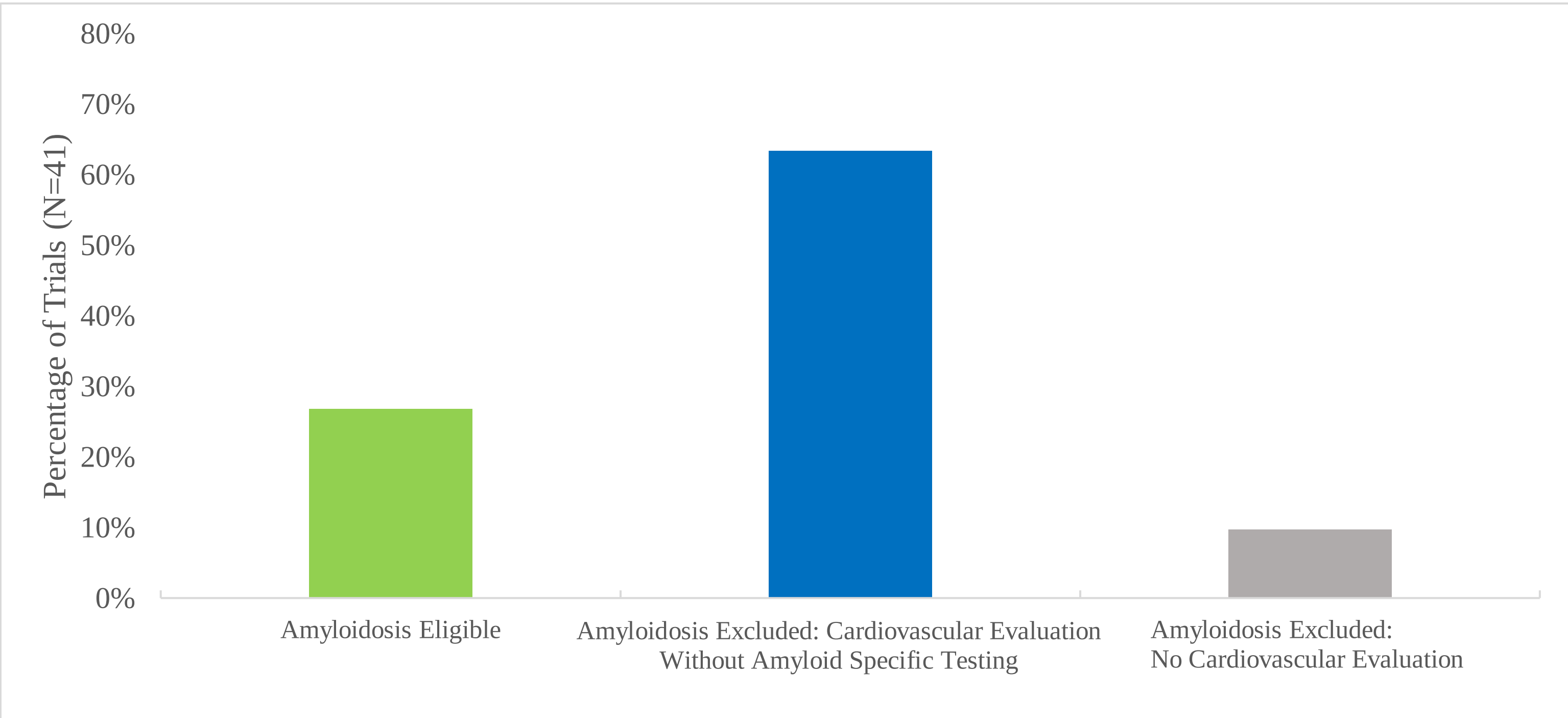

Supplement: Supplementary file 1 [file openhrt-13-2-s001.pdf]
